# The Comparison of Clinical Outcomes in Elderly (≥75 Years) and Non-Elderly (<75 Years) Patients with Acute Cholangitis Due to Choledocholithiasis

**DOI:** 10.3390/medicina59122171

**Published:** 2023-12-14

**Authors:** Tae-Yoon Lee, Sang-Hoon Lee, Young-Koog Cheon, Joon-Ho Wang

**Affiliations:** 1 Department of Internal Medicine, Konkuk University Hospital, Konkuk University School of Medicine, Seoul 05030, Republic of Korea; widebrow@empal.com (T.-Y.L.); lshjjang_2000@hanmail.net (S.-H.L.); yksky001@hanmail.net (Y.-K.C.); 2 Department of Internal Medicine, Konkuk University Chungju Hospital, Konkuk University School of Medicine, Chungju 27376, Republic of Korea

**Keywords:** acute cholangitis, prognosis, aging

## Abstract

*Background and Objectives*: Acute cholangitis may be fatal, particularly in elderly patients. According to the Tokyo Guidelines 2018, those aged ≥75 years are classified as moderate (Grade II) severity. However, it has not been established whether age itself is the deciding factor of poor outcomes. We studied the impact of old age (≥75 years) on the mortality and morbidity of acute cholangitis due to choledocholithiasis. *Materials and Methods*: We retrospectively examined 260 patients with calculous acute cholangitis who had undergone biliary drainage. Patients were divided into two groups: elderly (≥75 years) and non-elderly (<75 years). We aimed to compare organ dysfunction, in-hospital mortality, intensive care unit (ICU) hospitalization, and the severity of acute cholangitis. *Results*: Of 260 patients, 134 (51.5%) were in the elderly group and 126 (48.5%) were in the non-elderly group. The mean age was 72.3 ± 14.4 years, and 152 (58.5%) were men. The elderly patients showed a higher incidence of shock (12.7% vs. 4.8%, *p* = 0.029), respiratory dysfunction (7.5% vs. 0%, *p* = 0.002), and renal dysfunction (8.2% vs. 0.8%, *p* = 0.006) than the non-elderly patients. The overall in-hospital mortality rate was 2.7%, with no significant differences between the elderly and the non-elderly (4.5% vs. 0.8%, *p* = 0.121). The incidence of severe acute cholangitis was significantly higher in the elderly group (26.9% vs. 9.5%, *p* < 0.001). However, there was no significant difference in the rates of ICU hospitalization (9.7% vs. 4%, *p* = 0.088) and lengths of hospital stay (LOS) (8.3 d vs. 7.1 d, *p* = 0.086). *Conclusions*: No difference was observed in the in-hospital mortality, ICU hospitalization, or LOS between the elderly (≥75 years) and the non-elderly (<75 years) with calculous acute cholangitis. However, severe acute cholangitis was significantly more frequent in elderly patients.

## 1. Introduction

Acute cholangitis is a medical condition that presents with fever, jaundice, and right upper quadrant pain caused by stasis and infection in the bile duct [1]. It can range from mild cases that are resolved with medical treatment to a severe form that can lead to sepsis and septic shock [2]. The prognosis for acute cholangitis is good in most cases. However, acute cholangitis can become severe in elderly patients due to the combined effects of degenerative changes in the gastrointestinal system, concurrent medical conditions, medications, and the instability of the cardiovascular system [3]. Age is used to categorize the severity, with those aged 75 years or above being classified as moderate (Grade II) severity following the Tokyo Guidelines 2018 (TG18) [4].

Despite this, it is necessary to compare disease severity and clinical outcomes between elderly and non-elderly patients with acute cholangitis to establish a customized treatment strategy for elderly patients because it remains controversial whether age itself is the deciding factor in adverse outcomes for acute cholangitis. Recent studies from China and Turkey found no difference in mortality rates between elderly (≥80 years) and non-elderly (<80 years) patients with acute cholangitis [5,6]. However, a study from Turkey found that the mortality rate for those with acute cholangitis over 80 years of age was significantly higher than that of those younger than 80 [3]. Therefore, this study aimed to evaluate the effect of old age (≥75 years) used for severity grading in the TG18 on morbidity and mortality in acute cholangitis due to choledocholithiasis.

## 2. Materials and Methods

### 2.1. Patients and Data Collection

This study was carried out at Konkuk University Medical Center, Seoul and Chungju, Korea. We retrospectively investigated 260 patients with acute cholangitis due to choledocholithiasis who had undergone biliary drainage during hospitalization between April 2018 and May 2023. Those with acute cholangitis caused by hepatobiliary malignancy were excluded because malignant etiology is more frequently seen in elderly patients and results in poor prognosis. Age over 75 years was included as a risk factor in the TG18 for moderate acute cholangitis; thus, we classified the elderly as those aged 75 or over. The non-elderly were classified as those younger than 75. The laboratory and clinical characteristics of the two groups were compared. Demographic (age and sex), clinical, and procedural information was obtained from the electronic medical records. The clinical information collected on admission included the results of blood culture and laboratory results (total white blood cell (WBC) count, platelet count, prothrombin time international normalized ratio (PT-INR), C-reactive protein (CRP), total bilirubin, albumin, and creatinine). Upon admission, the Sequential Organ Failure Assessment (SOFA) score was calculated [7]. Procedural information included the method and timing of biliary drainage.

All patients were administered intravenous antibiotics, including a third-generation cephalosporin, piperacillin/tazobactam, ciprofloxacin, or carbapenem, after blood culture upon diagnosis of acute cholangitis. Crystalloid solutions were used for fluid resuscitation. When hypotension persisted despite proper hydration, an inotropic agent was administered. The attending physician determined the method (endoscopic retrograde cholangiopancreatography (ERCP) or percutaneous transhepatic biliary drainage (PTBD)) and the timing of biliary decompression.

To avoid any confounding effect on acute cholangitis, we excluded patients with accompanying acute inflammatory diseases (e.g., liver abscess, acute biliary pancreatitis, pneumonia, or pathologic diagnosis of acute cholecystitis in subsequent cholecystectomy) upon admission and during hospitalization. We excluded patients under 18 years of age and those with missing data. This study was approved by the Institutional Review Board of the hospital, and the requirement for informed consent was waived.

### 2.2. Study Outcomes and Definitions

We aimed to investigate the impact of old age (≥75 years) on in-hospital mortality and morbidity (the severity of acute cholangitis, details of organ dysfunction, and positive blood culture) in patients with acute cholangitis due to choledocholithiasis.

All participants in this study met the diagnostic criteria for acute cholangitis following the TG18 [4]. The severity of acute cholangitis was defined in accordance with the TG18. Severe acute cholangitis was defined as at least one impairment in the cardiovascular, neurological, respiratory, renal, hepatic, or hematologic systems. At admission and during hospitalization, shock was defined as hypotension that necessitated the use of dopamine ≥5 µg/kg or any dose of norepinephrine.

The association of old age with death, severe acute cholangitis, organ dysfunction, ICU admission, and positive blood culture was examined. In addition, we identified risk factors predictive of mortality in patients with acute cholangitis due to choledocholithiasis. The drainage time was defined as the time (hours) from the hospital admission to the end of biliary drainage procedures such as ERCP or PTBD. 

### 2.3. Statistical Analysis

SPSS for Windows 29.0 software (SPSS Inc., Chicago, IL, USA) was used for statistical analysis. The Kolmogorov–Smirnov test was used to evaluate the normality of the quantitative data distribution. Descriptive statistics for continuous and categorical variables are expressed in the data as the mean ± standard deviation and values (%), respectively. Chi-square and Fisher’s exact tests were employed to assess differences between categorical variables, while Student’s *t*-test was used for continuous variables with a normal distribution. For skewed-distribution variables, the median and IQR are presented, and the Mann–Whitney U test was used for comparison. To determine parameters for predicting in-hospital mortality, multivariate regression analysis was performed, using variables with a *p*-value < 0.05 in the univariate analysis. We selected the SOFA score for multivariate analysis to avoid multicollinearity because of the significant correlation between severe acute cholangitis, ICU admission, and the SOFA score. A *p*-value < 0.05 indicated statistical significance.

## 3. Results

### 3.1. Patient Demographics and Clinical Profiles

**Figure 1:**
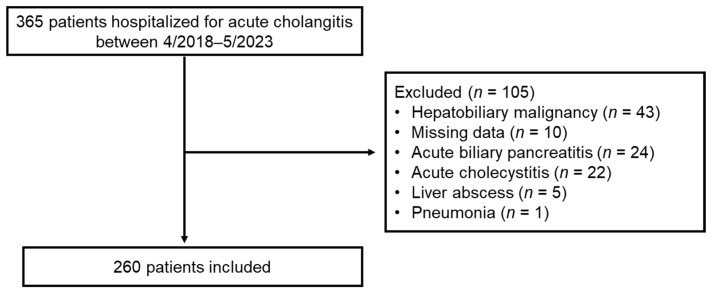
Flowchart of patient enrollment.

The flowchart of this study is shown in Figure 1. Among 365 hospitalized patients with acute cholangitis, 260 met the inclusion criteria and 105 were excluded. Forty-three patients were excluded because of underlying hepatobiliary malignancy. We excluded ten patients with missing data on variables required to calculate the SOFA score.

The demographic and clinical features of the elderly and non-elderly patients are listed in Table 1. Of the 260 patients, 134 (51.5%) were in the elderly group and 126 (48.5%) were in the non-elderly group. The mean age of the entire group was 72.3 years, with male predominance (*n* = 152, 58.5%). Among the 260 acute cholangitis patients, 100 (38.5%) had mild, 112 (43.1%) moderate, and 48 (18.5%) severe acute cholangitis. 

Biliary drainage was successful in all 260 patients. ERCP (*n* = 233; 89.6%) was the most commonly used procedure, followed by PTBD (*n* = 27; 10.4%). No patient underwent surgical drainage. Of 233 patients who underwent ERCP, the common bile duct stones were completely removed in 231 (99.1%), with 205 (87.9%) cases successful at the first attempt. Endoscopic biliary stenting was performed in 28 (12%) of 233 patients with incomplete stone extraction at the first ERCP. More than one ERCP was performed in twenty-six (11.1%) patients, of whom twenty-two (9.4%) required a second ERCP and four (1.7%) a third ERCP. Stone removal finally failed in two (0.8%) patients who could only undergo biliary stenting at the first ERCP because of hypoxia and severe tachycardia. An additional procedure for stone removal was not possible in either patient because of clinical deterioration. Of 27 patients who initially underwent PTBD, 11 underwent ERCP, which successfully removed the stones. Nine patients underwent percutaneous transhepatic cholangioscopy with or without electrohydraulic lithotripsy, and six patients underwent PTBD using the balloon sphincteroplasty flushing technique for stone removal. One patient could not undergo a further procedure after PTBD because of a poor general condition.

The elderly group had a significantly higher rate of severe disease (Grade III) at admission than the non-elderly group (26.9% vs. 9.5%, respectively, *p* < 0.001). The method (ERCP vs. PTBD) and timing of biliary drainage after admission were similar in both groups. Positive blood culture (*n* = 82, 31.5%) was similarly observed between the elderly and the non-elderly groups (35.1% vs. 27.8%, *p* = 0.285), and *E. coli* was the most common pathogen (*n* = 53/82, 64.6%).

Regarding inflammatory markers, the elderly group had a significantly higher WBC count (11,200 vs. 9880, *p* = 0.001) and NLR (12.81 vs. 8.72, *p* = 0.011) than the non-elderly group. However, the CRP level was similar between the two groups (7.96 vs. 6.26, *p* = 0.055). PT-INR was significantly prolonged in the elderly group (1.17 vs. 1.01, *p* < 0.001), and the platelet, albumin, and total bilirubin levels were significantly higher in the non-elderly group. Non-elderly patients were also more likely to undergo index admission cholecystectomy than non-elderly patients (36.5% vs. 20.1%, *p* = 0.004). 

### 3.2. Clinical Outcomes

Of forty-eight patients with severe acute cholangitis, thirty had one organ/system dysfunction and eighteen had two or more dysfunctions: cardiovascular dysfunction in twenty-three cases, hematologic dysfunction in nineteen, renal dysfunction in twelve, respiratory dysfunction in eleven, hepatic dysfunction in eight, and neurological dysfunction in four. Shock (12.7% vs. 4.8%, *p* = 0.029), respiratory dysfunction (7.5% vs. 0%, *p* = 0.002), and renal dysfunction (8.2% vs. 0.8%, *p* = 0.006) were significantly more common in the elderly group than in the non-elderly group. However, there was no significant difference between the elderly and the non-elderly groups in the rates of neurological, hepatic, or hematological dysfunction (Table 2).

Seven patients (2.7%) died within 30 days of hospitalization. Of the six deceased patients who had undergone PTBD, two patients had hypoxemia and shock, leading to a high risk for ERCP with conscious sedation, one patient was intubated due to respiratory failure, one patient had intrahepatic duct stones that were inaccessible by ERCP, one patient had esophageal stricture due to advanced thyroid cancer, and one patient had severe paralytic ileus that might have increased the risk of post-ERCP complications. One patient underwent ERCP with biliary stenting but died five days after the procedure because of pneumonia aggravation.

The in-hospital mortality showed no significant differences between the elderly and the non-elderly (4.5% vs. 0.8%, *p* = 0.121). ICU admission (*n* = 18, 6.9%) was required in 9.7% of the patients in the elderly group and 4% of those in the non-elderly group, with no significant difference (*p* = 0.121). The length of hospital stay (LOS) was not significantly different between the two groups (8.3 d vs. 7.1 d, *p* = 0.086). Elderly patients had a significantly higher SOFA score than non-elderly patients (3 (IQR, 2–4) vs. 2 (IQR, 2–3); *p* = 0.009).

### 3.3. Factors Predicting In-Hospital Mortality in the Entire Population

In univariate logistic analyses, in-hospital mortality was associated with severe acute cholangitis, PTBD as an initial biliary drainage method, positive blood culture, ICU admission, prolonged LOS, high SOFA score, leukocytosis, elevated CRP, prolonged PT-INR, and elevated creatinine levels.

A multivariate analysis controlling for related variables revealed that PTBD (odds ratio (OR) 20.27, *p* = 0.036), prolonged LOS (OR 1.15, *p =* 0.032), high SOFA score (OR 2.28, *p* = 0.015), and elevated creatinine (OR 1.96, *p* = 0.025) were significant predictors of in-hospital mortality (Table 3).

## 4. Discussion

This study divided patients with acute cholangitis caused by choledocholithiasis into two groups, elderly (≥75 years old) and non-elderly (<75 years old), and evaluated their clinical features and outcomes. Elderly patients with acute cholangitis had similar LOS and rates of in-hospital mortality and ICU admission compared to non-elderly patients. However, the rate of severe acute cholangitis was significantly higher in elderly patients than in non-elderly patients. Shock, respiratory dysfunction, and renal dysfunction were significantly more prevalent among the elderly than among the non-elderly.

Life expectancy has risen worldwide, resulting in an increase in the elderly population, who are more likely to suffer from gallstones and biliary events, including acute cholangitis, than the non-elderly [8]. Elderly patients with acute cholangitis are more prone than non-elderly patients with acute cholangitis to multiple comorbidities, sarcopenia, functional impairment, and limited capacity to endure stress, necessitating appropriate treatment plans [9]. Accordingly, age is often used in severity classification as a surrogate marker for comorbidities and functional capacity [5]; thus, those aged 75 years or over are classified as Grade II (moderate) severity as per the TG18 [4]. However, the number of studies on the prognosis of acute cholangitis in patients aged 75 years or older is limited [3,6,10,11]. In addition, studies explicitly comparing the clinical outcomes of acute cholangitis due to choledocholithiasis are rare [5].

In agreement with previous studies, this study revealed that while the elderly patients had a much more severe disease than the non-elderly patients [3,12], the percentage of in-hospital mortality and ICU admission in the elderly group was not significantly higher than that in the non-elderly group [5,6]. The prognosis of elderly patients with acute cholangitis is expected to be poorer than that of younger patients, similar to most other severe illnesses, because the association of advanced age with mortality in patients with acute cholangitis has already been demonstrated in several studies [3,13,14]. A recent study among 300 patients with acute cholangitis reported that the mortality rate was higher in the elderly group (≥80 years old) than in the non-elderly group (10.4% vs. 5.9%, *p* = 0.045) [3]. Because fatal outcomes are more likely for severe cholangitis, as defined by the presence of organ failure, the results we provided were not what was typically expected. In this study, the lower rates of in-hospital mortality and ICU admission, combined with the small sample size, may have prevented us from finding a significant difference between the two groups. However, several studies have reported similar results as in the present study. For example, an international multicenter study similarly noted that old age (≥75 years) did not significantly correlate with 30-day mortality (OR 1.35 (95% CI 0.98–1.88)) [11]. In addition, recent studies from China and Turkey revealed that mortality rates in acute cholangitis patients did not differ between the elderly and the non-elderly [5,6]. These results indicate that developing surrogate markers of frailty other than age is essential to predict the prognosis of acute cholangitis more precisely before overt organ failure.

The mortality rate in our study was 2.7%, much lower than the 5–10% reported in other studies [15], but comparable to the 1.5% reported in the study by Park et al., which performed early ERCP in most patients (92.7%) [16]. The positive outcome in this study could be attributed to early biliary drainage being performed in most patients with acute cholangitis within 48 h. We treated 91.6% (238 out of 260) of the patients with early ERCP or PTBD. Another reason for the low mortality rate is that we excluded acute cholangitis resulting from malignancy because acute cholangitis due to malignant biliary obstruction has a poorer prognosis [17]. Acehan et al. reported that the malignant etiology was an independent risk factor for in-hospital mortality in patients with acute cholangitis [6]. Tsou et al. also reported that 30-day mortality was significantly higher in the malignant biliary obstruction group than in the choledocholithiasis group (5.4% vs. 0.7%, *p* = 0.045) among 516 patients with acute cholangitis [18].

Patients with signs of acute cholangitis should undergo an initial evaluation using the TG18 because this is the standard for the initial triage and treatment of these patients. Patients with Grade III (severe) disease and signs of organ failure generally require ICU admission and close observation. Therefore, it is crucial to investigate the difference in the pattern of organ dysfunction in detail between elderly and non-elderly patients. In this study, elderly patients showed a significantly higher incidence of shock (12.7% vs. 4.8%, *p* = 0.029), respiratory dysfunction (7.5% vs. 0%, *p* = 0.002), and renal dysfunction (8.2% vs. 0.8%, *p* = 0.006) than the non-elderly group. These three organ systems are used to calculate the modified Marshall organ failure score, which is based on the original Marshall score but focuses on three organ systems rather than six [19]. On the other hand, the rates of neurological, hepatic, and hematological dysfunction were similar between the elderly and the non-elderly groups. Similarly to the results of this study, Sugiyama et al. reported that elderly patients had higher rates of septic shock and renal dysfunction than non-elderly patients with acute cholangitis caused by choledocholithiasis [10]. Another Japanese study found that respiratory dysfunction had the highest odds ratio (OR 2.78 (95% CI 1.43–5.4)), but hepatic and hematological dysfunction had the lowest odds ratio in predicting 30-day mortality [11]. 

Of the numerous parameters related to in-hospital mortality in the univariate analysis, PTBD, prolonged LOS, high SOFA score, and elevated creatinine levels were significant predictors of in-hospital mortality in the multivariate analysis. In this study, six (85.7%) of the seven patients who died had undergone PTBD as the initial drainage method. While ERCP requires moderate sedation or general anesthesia, PTBD can be performed using local anesthesia. Given that all deaths had been categorized as severe acute cholangitis at admission, these patients had a greater risk of adverse reactions to sedation, and in this study, three patients had hypoxemia. PTBD is also useful in cases of gastrointestinal obstruction or intrahepatic duct stones, and in this study, one deceased patient had esophageal obstruction, one had intrahepatic duct stones, and one had severe paralytic ileus. Therefore, the attending physician may have preferred PTBD because it is considered to be less invasive and does not require moderate sedation/general anesthesia [5]. Consistently with previous studies [20,21], elevated creatinine levels were associated with acute cholangitis mortality in our study. Elsewhere, a French study reported that the SOFA score (OR 1.14, 95% CI 1.05–1.24) was associated with the in-hospital mortality of patients admitted to the ICU with acute cholangitis [22].

The primary strength of this study is in providing the first detailed exploration of the differences in the dysfunction of six organ systems between the elderly and the non-elderly. Shock, respiratory dysfunction, and renal dysfunction were significantly more frequent in the elderly group compared to the non-elderly group. Secondly, we chose strict inclusion criteria that excluded concurrent acute inflammatory diseases, such as acute cholecystitis or biliary pancreatitis, to avoid any confounding effect on acute cholangitis. However, our study has several limitations. First, it was a retrospective study, with a relatively small number of patients. Therefore, this study may be underpowered to make definitive conclusions on the association of old age with the prognosis of acute cholangitis. Furthermore, it might have inherent biases due to the single-center setting. Finally, it is limited by missing data.

## 5. Conclusions

Despite the greater prevalence of severe acute cholangitis in the elderly when compared with the non-elderly, in-hospital mortality, ICU admission, and LOS did not differ between elderly and non-elderly patients with acute cholangitis due to choledocholithiasis. Thus, the outcomes of acute cholangitis may not be accurately predicted by age alone. However, a large prospective trial is required to confirm this finding.

## Figures and Tables

**Table 1:** Demographic and clinical profiles of 260 patients diagnosed with acute calculous cholangitis.

	Overall (*n* = 260)	Elderly (*n* = 134)	Non-Elderly (*n* = 126)	*p*-Value
Age, mean ± SD, years	72.3 ± 14.4	83.5 ± 5.3	60.6 ± 11.4	<0.001
Male, gender, *n* (%)	151 (58.1%)	60 (44.8%)	92 (73%)	<0.001
TG 18 severity grading				<0.001
Mild	100 (38.5%)	25 (18.7%)	75 (59.5%)	
Moderate	112 (43.1%)	73 (54.5%)	39 (31%)	
Severe	48 (18.5%)	36 (26.9%)	12 (9.5%)	
Biliary drainage method				0.229
ERCP	233 (89.6%)	117 (87.3%)	116 (92.1%)	
PTBD	27 (10.4%)	17 (12.7%)	10 (7.9%)	
Timing of biliary drainage				0.806
Within 24 h	184 (70.8%)	93 (69.4%)	91 (72.2%)	
From 24 to 48 h	54 (20.8%)	30(22.4%)	24 (19%)	
After 48 h	22 (8.5%)	11 (8.2%)	11 (8.7%)	
Positive blood culture	82 (31.5%)	47 (35.1%)	35 (27.8%)	0.285
Gram-positive	7 (2.7%)	4 (3%)	3 (2.4%)	
Gram-negative	75 (28.8%)	43 (32.1%)	32 (25.4%)	
Laboratory data				
WBC count (/µL)	10,495 (7880–13,535)	11,200 (8295–14,792)	9880 (7100–12,432)	0.001
NLR	9.84 (5.72–19.18)	12.81 (6.84–20.82)	8.72 (4.76–17.05)	0.011
CRP (mg/dL)	7.14 ± 7.15	7.96 ± 7.15	6.26 ± 7.07	0.055
Platelet (×10^3^/µL)	207.08 ± 90.42	195.63 ± 93.39	219.27 ± 85.84	0.032
PT-INR	1.09 ± 0.32	1.17 ± 0.41	1.01 ± 0.16	<0.001
Creatinine (mg/dL)	1.04 ± 0.84	1.09 ± 0.86	0.98 ± 0.81	0.29
Albumin (g/dL)	3.81 ± 0.46	3.62 ± 0.41	4.01 ± 0.42	<0.001
Total bilirubin (mg/dL)	3.08 (2.01–4.75)	2.82 (1.59–4.22)	3.28 (2.23–5.76)	<0.001
Index cholecystectomy	73 (28.1%)	27 (20.1%)	46 (36.5%)	0.004

Values shown are means ± SD or medians (25th–75th percentiles). SD, standard deviation; TG 18, Tokyo Guidelines 2018; ERCP, endoscopic retrograde cholangiopancreatography; PTBD, percutaneous transhepatic biliary drainage; WBC, white blood cell; NLR, neutrophil–lymphocyte ratio; CRP, C-reactive protein; PT-INR, prothrombin time international normalized ratio.

**Table 2:** Clinical outcomes between elderly and non-elderly patients.

	Overall (*n* = 260)	Elderly (*n* = 134)	Non-Elderly (*n* = 126)	*p*-Value
Organ/system dysfunction				
Shock	23 (8.8%)	17 (12.7%)	6 (4.8%)	0.029
Neurological	4 (1.5%)	4 (3%)	0 (0%)	0.123
Respiratory	10 (3.8%)	10 (7.5%)	0 (0%)	0.002
Renal	12 (4.6%)	11 (8.2%)	1 (0.8%)	0.006
Hepatic	8 (3.1%)	7 (5.2%)	1 (0.8%)	0.067
Hematological	19 (7.3%)	13 (9.7%)	6 (4.8%)	0.155
In-hospital mortality	7 (2.7%)	6 (4.5%)	1 (0.8%)	0.121
ICU admission	18 (6.9%)	13 (9.7%)	5 (4%)	0.088
Length of hospital stay, days	7.7 ± 5.6	8.3 ± 5.8	7.1 ± 5.3	0.086
SOFA score	2 (2–3)	3 (2–4)	2 (2–3)	0.009

ICU, intensive care unit; SOFA, Sequential Organ Failure Assessment.

**Table 3:** Factors predicting in-hospital mortality in patients with acute calculous cholangitis.

			Univariate Analysis		Multivariate Analysis	
	Alive (*n* = 253)	Death (*n* = 7)	OR (95% CI)	*p*-Value	OR (95% CI)	*p*-Value
Age, mean ± SD, years	72.1 ± 14.4	83 ± 8.6	1.08 (0.99–1.18)	0.121		
Male, gender, *n* (%)	147 (58.1%)	5 (71.4%)	0.55 (0.1–2.91)	0.703		
Severe acute cholangitis (%)	41 (16.2%)	7 (100%)	2.29 (1.51–3.06)	<0.001		
Biliary drainage method						
ERCP	232 (91.7%)	1 (14.3%)	Ref.			
PTBD	21 (8.3%)	6 (85.7%)	19.38 (1.06–35.42)	0.046	20.27 (1.22–33.63)	0.036
Delayed biliary drainage (after 48 h)	20 (7.9%)	2 (28.6%)	1.07 (0.94–1.23)	0.161		
Positive blood culture	76 (30%)	6 (85.7%)	13.97 (1.65–46.55)	0.005	3.69 (0.07–7.78)	0.544
ICU admission	11 (4.3%)	7 (100%)	23.52 (3.86–55.57)	<0.001		
Length of hospital stay	7.39 ± 4.52	20.14 ± 18.16	1.24 (1.1–1.39)	<0.001	1.15 (1.06–1.23)	0.032
SOFA score	2 (2–3)	9 (8–13)	3.21 (1.24–8.26)	<0.001	2.28 (1.54–3.39)	0.015
Index cholecystectomy	73 (28.9%)	0 (0%)	0.96 (0.93–0.99)	0.196		
Laboratory data						
WBC count (/µL)	11,164 ± 5215	11,164 ± 5215	1.05 (1.01–1.1)	0.011	1 (0.99–1.01)	0.645
NLR	15.67 ± 16.4	15.68 ± 9.71	0.97 (0.91–1.04)	0.998		
CRP (mg/dL)	6.94 ± 6.96	14.42 ± 10.16	1.09 (1.01–1.19)	0.006	1.03 (0.94–1.14)	0.476
Platelet (×10^3^/µL)	208.83 ± 90.27	144 ± 76.81	0.98 (0.97–1)	0.056		
PT-INR	1.01 (0.94–1.11)	1.32 (1.13–1.85)	3.59 (1.05–12.2)	0.004	2.99 (0.12–7.39)	0.5
Creatinine (mg/dL)	0.81 (0.67–1.06)	1.82 (0.92–4.97)	2.3 (1.1–4.8)	0.004	1.96 (1.24–3.1)	0.025
Albumin (g/dL)	3.7 ± 0.45	3.47 ± 0.62	0.25 (0.02–2.3)	0.224		
Total bilirubin (mg/dL)	3.08 (2.01–4.68)	3.98 (0.88–7.31)	1.08 (0.78–1.47)	0.632		

Values shown are means ± SD or medians (25th–75th percentiles). SD, standard deviation; ERCP, endoscopic retrograde cholangiopancreatography; PTBD, percutaneous transhepatic biliary drainage; ICU, intensive care unit; SOFA, Sequential Organ Failure Assessment; WBC, white blood cell; NLR, neutrophil–lymphocyte ratio; CRP, C-reactive protein; PT-INR, prothrombin time international normalized ratio.

## Data Availability

The datasets generated for this study are available on request to the corresponding author.
